# Indication for Antibiotic Prescription Among Children Attending Primary Healthcare Services in Rural Burkina Faso

**DOI:** 10.1093/cid/ciab471

**Published:** 2021-05-21

**Authors:** Ali Sié, Mamadou Ouattara, Mamadou Bountogo, Clarisse Dah, Guillaume Compaoré, Valentin Boudo, Elodie Lebas, Jessica Brogdon, Fanice Nyatigo, Benjamin F Arnold, Thomas M Lietman, Catherine E Oldenburg

**Affiliations:** 1 Centre de Recherche en Santé de Nouna, Nouna, Burkina Faso; 2 Francis I. Proctor Foundation, University of California, San Francisco, San Francisco, California, USA; 3 Department of Ophthalmology, University of California, San Francisco, San Francisco, California, USA; 4 Department of Epidemiology & Biostatistics, University of California, San Francisco, San Francisco, California, USA

**Keywords:** antibiotics, pneumonia, malaria, diarrhea, antibiotic stewardship

## Abstract

Of 61 355 visits by children <5 years old to 48 government-run primary healthcare facilities in Nouna District, Burkina Faso, 30 975 had an antibiotic prescribed (58% for pneumonia diagnoses). A minority of prescriptions were for diagnoses not requiring antibiotics, including malaria, nonbloody diarrhea, and cough without pneumonia.

Antibiotic consumption can select for resistant organisms at both individual and community levels [1, 2]. Reducing unnecessary antibiotic use may reduce antibiotic consumption without compromising necessary antibiotic use. In regions without diagnostic laboratory facilities, including in many areas of rural sub-Saharan Africa, pediatric antibiotic treatment is guided by clinical symptoms [3, 4]. For example, the World Health Organization (WHO) recommends that children with fast breathing and/or chest indrawing pneumonia receive oral amoxicillin, whereas those with a cough and cold only (no pneumonia) should not receive antibiotics [5, 6]. Similarly, WHO recommends antibiotic treatment for children with bloody diarrhea (dysentery) but not for nondysenteric diarrhea. In the current study, we used routinely collected data from primary healthcare facilities in rural Burkina Faso to evaluate antibiotic prescription among children <5 years of age with common childhood illnesses diagnosed and identify diagnoses with the most potential to reduce unnecessary antibiotic use.

## METHODS

### Study Setting

This study took place in 48 primary health care centers (Centres de Santé et de Promotion Sociale [CSPS]) in Nouna District, Burkina Faso [7]. Nouna District is a rural area of northwestern Burkina Faso that experiences highly seasonal rainfall from approximately July to October [8]. The high malaria transmission season coincides with the rainy season, when overall healthcare use is typically higher but antibiotic prescriptions are proportionally lower, because more diagnoses are due to malaria [8]. Healthcare in the study area for children <5 years of age is provided free of charge by the government. The CSPS represents the first level of healthcare available in the healthcare system, offering basic preventive and curative care, such as routine vaccinations and antenatal care. These facilities do not typically have access to diagnostic laboratory facilities, with diagnostic testing limited to rapid diagnostic tests (RDT) for malaria.

### Data Collection

We extracted data from all sick child visits from March to November 2020 at each facility, for all children <5 years of age. Data were restricted only to first-time visits, and we excluded follow-up visits in our analysis. We extracted data from written ledgers issued by the Ministry of Health for recording diagnostic and treatment information, which were entered into a custom-made electronic data capture form. We included information on the date of the visit, the child’s age and sex, diagnosis, and treatment. We focused the analysis on the most commonly diagnosed infections and infectious symptoms, including pneumonia, malaria, diarrhea, fever, and cough. 

When RDTs are available, they are used to diagnose malaria. Because RDT stockouts are common, malaria is diagnosed symptomatically when RDTs are not available. All other diagnoses are made following WHO community management of childhood illness algorithms, although the accuracy of these diagnoses is not captured in the data set. We categorized diagnoses as mutually exclusive categories, including pneumonia; dysentery without pneumonia; malaria without pneumonia or dysentery; nonbloody diarrhea without dysentery, pneumonia, or malaria; fever without pneumonia, malaria, nonbloody diarrhea, or dysentery; cough without pneumonia, dysentery, malaria, nonbloody diarrhea, or fever; and all other diagnoses. excluding the previous categories.

### Data Analysis

We calculated the proportion of each major diagnosis that received an antibiotic prescription overall and by antibiotic (including amoxicillin/penicillin, erythromycin, and cotrimoxazole), and corresponding binomial confidence intervals account for clustering at the CSPS level, using a nonparametric bootstrap. We then calculated the prevalence ratio of an antibiotic’s being prescribed for children with each diagnosis, versus pneumonia as the reference category, using a modified Poisson model [9] and adjusting for the child’s age and sex and the season of the visit, with standard errors adjusted for clustering at the CSPS level (Huber-White robust standard errors). All analyses were conducted using Stata software, version 15.1 (StataCorp).

## RESULTS

Of 61 355 visits, 30 975 (50.5%) received an antibiotic prescription. Antibiotic prescriptions were more common in the dry season than during the rainy season (in 57.1% vs 42.9% of visits; Supplementary Table 1). Of all antibiotic prescriptions, 23 554 (76%) were associated with pneumonia, malaria, diarrhea, dysentery, fever, or cough. The majority of the remaining antibiotic prescriptions were primarily for skin conditions (Supplementary Table 2).

**Figure 1.**
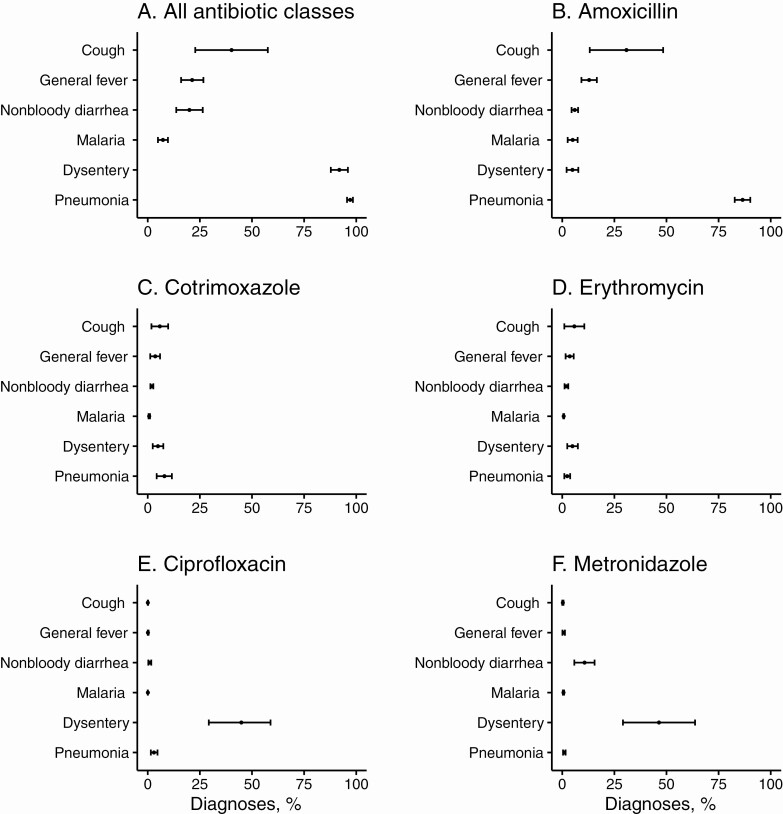
Percentage of each major infectious diagnosis receiving an antibiotic prescription, overall (*A*) and by specific antibiotic, including amoxicillin (*B*), cotrimoxazole (*C*), erythromycin (*D*), ciprofloxacin (*E*), and metronidazole (*F*). Dots represent point estimates; bars, exact binomial 95% confidence intervals.

The majority of pneumonia and dysentery diagnoses received an antibiotic prescription (97.1% and 91.9%, respectively; Figure 1). Amoxicillin was the most commonly prescribed antibiotic for pneumonia, and metronidazole and ciprofloxacin were commonly prescribed for dysentery (Figure 1). Antibiotics were prescribed for 7.3% of malaria diagnoses, and malaria-only diagnoses were significantly less likely to receive an antibiotic prescription compared with pneumonia (Supplementary Table 3). However, given the frequency of malaria diagnosis, 1462 antibiotic prescriptions were given for malaria diagnoses without pneumonia (approximately 5% of all antibiotic prescriptions). Approximately 1 in 5 nonbloody diarrhea diagnoses (20.0%) received an antibiotic prescription, mostly for ciprofloxacin (14.9% of nonbloody diarrhea diagnoses). Pneumonia diagnoses were responsible for the majority of antibiotic prescriptions (Supplementary Figure 1) and were significantly more likely than other diagnoses to receive an antibiotic prescription (Supplementary Table 3).

## DISCUSSION

We document that the majority of antibiotic prescriptions among children attending primary care facilities in Nouna District are dispensed for pneumonia diagnoses. Pneumonia is the leading cause of childhood death, and treatment is based on WHO-recommended treatment classification. Childhood pneumonia can have viral or bacterial etiology, but distinguishing between viral and bacterial pneumonia requires sophisticated diagnostic facilities that many children in regions with high pneumonia burden do not have access to. Because nonsevere pneumonia cases may be self-limiting and viral, recent studies have evaluated whether antibiotics are necessary for mild pneumonia without danger signs [10–12]. Although most cases of nonsevere pneumonia recover without antibiotics, the proportion of cases recovered after 4 days was slightly higher with use of amoxicillin than without in randomized controlled trials [10, 11]. The majority of antibiotic prescribing in the current study was appropriate, given the pneumonia diagnosis, because antibiotics are indicated for pneumonia. However, the diagnostic accuracy of pneumonia in this setting is unknown, and improvements in pneumonia diagnosis may reduce antibiotic consumption.

Malaria diagnoses were uncommonly prescribed an antibiotic. Amoxicillin, the antibiotic most commonly prescribed overall and for malaria, does not have antimalarial properties. The WHO does not recommend routine antibiotic treatment for uncomplicated malaria. Pneumonia and malaria can present with similar clinical signs, including fever, which often results in diagnosis and treatment for both conditions. Previous analyses have shown that while concomitant diagnosis is common, true overlap in disease is rare [13]. Primary healthcare facilities in the study area have RDT capability for diagnosing malaria but do not typically have radiographic facilities for diagnosing pneumonia. An improvement in diagnostics for pneumonia could reduce unnecessary antibiotic treatment if pneumonia is misdiagnosed .

WHO guidelines recommend antibiotic treatment for dysentery, with ciprofloxacin recommended as first-line treatment, as such cases are presumed to be due to *Shigella* spp. [6]. In the study area, dysentery presenting with mucus is presumed to be amoebiasis (*Entamoeba histolytica*), and metronidazole treatment is prescribed. In the current analysis, approximately half of antibiotic prescriptions for dysentery were metronidazole. *E. histolytica* is a relatively uncommon cause of childhood diarrhea in other settings in the Sahel [14], and it is possible that the current classification system is overcalling amoebic dysentery. Etiologic studies of diarrhea in this setting may be useful for determining the most common causes of diarrhea in these children and guiding treatment decisions. Although the WHO does not recommend antibiotic treatment for nonbloody diarrhea, studies have shown that it is common in multiple settings [15, 16]. In the present study, approximately 1 in 5 cases of nonbloody diarrhea were treated with antibiotics, which may represent an opportunity to reduce antibiotic prescription.

Routinely collected healthcare surveillance data have inherent limitations that must be considered. Although we were able to rapidly collect data on thousands of child visits in the study area, these are data are subject to misclassification. If entered incorrectly in ledgers, diagnosis and treatment data could be misclassified. In the absence of laboratory diagnostic facilities, diagnoses themselves may be misclassified. RDT stockouts are not uncommon, and in the absence of an available RDT malaria diagnoses are made symptomatically. Similarly, although the goal is to follow WHO algorithms for diagnosis of pneumonia, in practice they are not always followed. We did not have data on RDT stockouts, on which malaria diagnoses were made based on symptoms versus with an RDT, or on the quality of the pneumonia diagnoses. 

Data were not prospectively collected specifically for the purposes of this study, and thus we have limited information on copresenting symptoms that may drive treatment decisions and the accuracy of diagnoses. Data were collected in a single district of northwestern Burkina Faso and may not be generalizable outside of the region. Despite these limitations, our data represent real-world diagnostic and treatment decisions based on currently available infrastructure in primary healthcare facilities in a rural area of Burkina Faso, and they data offer insight into treatment decisions made under real-world conditions.

These data demonstrate that the majority of pediatric antibiotic prescriptions in this area of Burkina Faso were due to a pneumonia diagnosis, with a minority of prescriptions due to diagnoses for which antibiotic use is not indicated, including malaria and nonbloody diarrhea. Interventions to reduce the use of antibiotics for nonindicated diagnoses may reduce overall antibiotic consumption.

## Supplementary Data

Supplementary materials are available at *Clinical Infectious Diseases* online. Consisting of data provided by the authors to benefit the reader, the posted materials are not copyedited and are the sole responsibility of the authors, so questions or comments should be addressed to the corresponding author.

ciab471_suppl_Supplemental_Material

## Notes

***Financial support.*** This work was supported by the Bill & Melinda Gates Foundation (grant OPP1187628 to institution to support parent study; article processing charges)

***Potential conflicts of interest.*** A. S. reports grants from the National Institute of Child Health and Development National Institutes of Health (NIH), and travel support from the Bill & Melinda Gates Foundation, outside the submitted work. M. B. reports travel support from the Bill & Melinda Gates Foundation, outside the submitted work. B. F. A. reports grants from the National Institute of Allergy and Infectious Diseases, NIH, and the Neglected Tropical Diseases (NTD ) Support Center, Task Force for Global Health; travel support from the Bill & Melinda Gates Foundation; and advisory board fees from the National Eye Institute, NIH, all outside the submitted work. T. M. L. reports grants from the National Eye Institute, NIH, and the NTD Support Center, Task Force for Global Health; and travel support from the Bill & Melinda Gates Foundation, outside the submitted work. C. E. O. reports research grants to support ongoing work, to her institution from the National Institute of Child Health and Development and the National Eye Institute, NIH, and travel support from the Bill & Melinda Gates Foundation. All other authors report no potential conflicts. All authors have submitted the ICMJE Form for Disclosure of Potential Conflicts of Interest. Conflicts that the editors consider relevant to the content of the manuscript have been disclosed.
