# Supplementary material for: Indication for Antibiotic Prescription Among Children Attending Primary Healthcare Services in Rural Burkina Faso
Source: Clin Infect Dis. 2021 May 21;73(7):1288–91. doi: 10.1093/cid/ciab471 (PMC8492132; doi:10.1093/cid/ciab471)
Supplement: ciab471_suppl_Supplemental_Material [file ciab471_suppl_supplemental_material.docx]

**Supplemental Material for: “Indication for antibiotic prescription among children attending primary healthcare services in rural Burkina Faso”**

**Table S1.** Descriptive characteristics of childhood primary healthcare visits (N=61,355)

|  | **Antibiotic Prescribed** | **No Antibiotic Prescribed** | ***P*-value** |
| --- | --- | --- | --- |
| Total visits, N (%) | 30,975 (50.5%) | 30,380 (49.5%) |  |
| Age, months, median (IQR) | 16 (8 to 29) | 24 (12 to 37) | <0.001 |
| Female sex, N (%) | 13,919 (44.9%) | 13,467 (44.3%) | 0.13 |
| Season |  |  |  |
| Rainy (July-October) | 13,281 (42.9%) | 15,953 (52.5%) | <0.001 |
| Dry (November-June) | 17,694 (57.1%) | 14,427 (47.5%) |  |

Abbreviations: IQR, interquartile range

**Table S2.** Other diagnoses not including pneumonia, malaria, non-bloody diarrhea, dysentery, general fever, or cough by antibiotic prescription (N=13,005 child visits). Categories are not mutually exclusive and thus may total more than 100%.

| **Diagnosis** | **Antibiotic Prescribed,**  **N (%)** | **No Antibiotic Prescribed,**  **N (%)** | **Total** |
| --- | --- | --- | --- |
| Eczema | 2,198 (76.1%) | 692 (23.9%) | 2,890 |
| Dermatological disease | 694 (84.0%) | 132 (16.0%) | 826 |
| Candidiasis | 109 (13.8%) | 684 (86.3%) | 793 |
| Severe malaria | 289 (43.5%) | 375 (56.5%) | 664 |
| Conjunctivitis | 611 (95.3%) | 30 (4.7%) | 641 |
| Wound | 338 (63.9%) | 191 (36.1%) | 529 |
| Dehydration | 31 (8.0%) | 355 (92.0%) | 386 |
| Intestinal parasitosis | 85 (23.9%) | 270 (76.1%) | 355 |
| Otitis | 232 (97.5%) | 6 (2.5%) | 238 |
| Urinary tract infection | 205 (95.4%) | 10 (4.7%) | 215 |
| Rhinitis | 101 (55.8%) | 80 (44.2%) | 181 |
| Gastrointestinal disorder | 94 (61.9%) | 58 (38.2%) | 152 |
| Cold | 75 (54.4%) | 63 (45.7%) | 138 |
| Chicken pox | 82 (64.1% | 46 (35.9%) | 128 |
| Otorhinolaryngological disease | 92 (97.9%) | 2 (2.1%) | 94 |
| Allergy | 28 (30.4%) | 64 (69.6%) | 92 |
| Severe acute malnutrition | 72 (80.0%) | 18 (20.0%) | 90 |
| Gastritis | 74 (88.1%) | 10 (11.9%) | 84 |
| Infant infection | 80 (95.2%) | 4 (4.8%) | 84 |
| Burn | 58 (74.4%) | 20 (25.6%) | 78 |
| Moderate acute malnutrition | 26 (34.7%) | 49 (65.3%) | 75 |
| Gastrointestinal disease | 35 (47.3%) | 39 (52.7%) | 74 |
| Bronchitis | 69 (100%) | 0 | 69 |
| Anemia | 23 (41.1%) | 33 (58.9%) | 56 |
| Urinary disease | 51 (94.4%) | 3 (5.6%) | 54 |
| Injury | 25 (61.0%) | 16 (39.0%) | 41 |
| Food poisoning | 29 (82.9%) | 6 (17.1%) | 35 |
| Gastrointestinal infection | 25 (80.7%) | 6 (19.4%) | 31 |
| Mastoiditis | 27 (96.4%) | 1 (3.6%) | 28 |
| Pharyngitis | 22 (78.6%) | 6 (21.4%) | 28 |
| Mumps | 11 (64.7%) | 6 (35.3%) | 17 |
| Vomiting | 7 (41.2%) | 10 (58.8%) | 17 |
| Diphtheria | 11 (68.8%) | 5 (31.3%) | 16 |
| Sting or bite | 5 (38.5%) | 8 (61.5%) | 13 |
| Respiratory disease | 10 (90.9%) | 1 (9.1%) | 11 |
| Umbilical hernia | 2 (18.2%) | 9 (81.8%) | 11 |
| Eye disease | 8 (80.0%) | 2 (20.0%) | 10 |
| Cavities and complications | 7 (100%) | 0 | 7 |
| Snake bite | 4 (57.1%) | 3 (42.9%) | 7 |
| Typhoid and paratyphoid fever | 6 (85.7%) | 1 (14.3%) | 7 |
| Dental disease | 3 (60%) | 2 (40%) | 5 |
| Rhinopharyngitis | 5 (100%) | 0 | 5 |
| Sexually transmitted infection | 5 (100%) | 0 | 5 |
| Avitaminosis A | 1 (25.0%) | 2 (75.0%) | 3 |
| Dog bite | 2 (66.7%) | 1 (33.3%) | 3 |
| Inguinale hernia | 1 (33.3%) | 2 (66.7%) | 3 |
| Lesion of gums and mucous membranes | 2 (66.7%) | 1 (33.3%) | 3 |
| Lesion of the lips and cheeks | 2 (66.7%) | 1 (33.3%) | 3 |
| Asthma | 1 (50%) | 1 (50%) | 2 |
| Bowel obstruction | 0 | 2 (100%) | 2 |
| Underweight | 2 (100%) | 0 | 2 |
| Anthrax | 1 (100%) | 0 | 1 |
| Bronchopneumonia | 1 (100%) | 0 | 1 |
| HIV | 0 | 1 (100%) | 1 |
| Hydrocele | 1 (100%) | 0 | 1 |
| Low pneumonia | 1 (100%) | 0 | 1 |
| Meningitis | 1 (100%) | 0 | 1 |
| Paralysis | 0 | 1 (100%) | 1 |
| Rabies | 1 (100%) | 0 | 1 |
| Sickle cell disease | 0 | 1 (100%) | 1 |
| Sinusitis | 1 (100%) | 0 | 1 |
| Sore throat | 1 (100%) | 0 | 1 |
| Tetanus | 0 | 1 (100%) | 1 |
| Yellow fever | 0 | 1 (100%) | 1 |
| Other | 2,429 (59.4%) | 1,660 (40.6%) | 4,089 |

**Table S3.** Percentage of each diagnosis receiving an antibiotic prescription and prevalence ratios for antibiotic receipt by diagnosis (N=61,355 healthcare encounters)

| **Diagnosis** | **Total** | **Antibiotic Prescribed,**  **N (%)** | **No Antibiotic Prescribed,**  **N (%)** | **Adjusted Prevalence Ratio^1^ (95% CI)** |
| --- | --- | --- | --- | --- |
| Pneumonia | 18,607 | 18,066 (97.1%) | 541 (2.9%) | Reference |
| Malaria^2^ | 19,941 | 1,462 (7.3%) | 18,479 (92.7%) | 0.08 (0.06 to 0.10) |
| Dysentery^3^ | 914 | 840 (91.9%) | 74 (8.1%) | 0.95 (0.91 to 0.99) |
| Non-bloody diarrhea^4^ | 3,474 | 695 (20.0%) | 2,778 (80.0%) | 0.20 (0.15 to 0.28) |
| General fever^5^ | 2,081 | 444 (21.3%) | 1,637 (78.7%) | 0.22 (0.17 to 0.29) |
| Cough^6^ | 3,334 | 1,341 (40.2%) | 1,993 (59.8%) | 0.41 (0.26 to 0.64) |
| All others^7^ | 13,005 | 8,127 (62.5%) | 4,878 (37.5%) | 0.64 (0.60 to 0.69) |
| Total | 61,355 | 30,975 (50.5%) | 30, 380 (49.5%) |  |

^1^Risk ratios comparing probability of receiving an antibiotic for that diagnosis vs pneumonia, adjusted for the child’s age and sex and season of the visit, with standard errors adjusted for clustering at the health facility level; ^2^Malaria with no pneumonia or dysentery diagnosis; ^3^Dysentery diagnosis with no pneumonia diagnosis; ^4^Non-bloody diarrhea with no pneumonia, malaria, or dysentery diagnosis; ^5^Fever with no pneumonia, malaria, dysentery, or non-bloody diarrhea diagnosis; ^6^Cough with no pneumonia, malaria, dysentery, non-bloody diarrhea, or fever diagnosis; ^7^Other diagnosis than pneumonia, malaria, dysentery, non-bloody diarrhea, general fever, or cough, other diagnoses are detailed in Table S2.

**Figure S1.** Percent of antibiotic prescriptions by diagnosis (N=30,975 antibiotic prescriptions). Prescribed antibiotics included amoxicillin (N=22,083 prescriptions), cotrimoxazole (N=2,396 prescriptions), erythromycin (N=3,610 prescriptions), ciprofloxacin (N=561 prescriptions), and metronidazole (N=1,538 prescriptions). Other antibiotic classes accounted for 787 prescriptions and are not depicted here.
